# Demographic change and urban health: Towards a novel agenda for delivering sustainable and healthy cities for all

**DOI:** 10.12688/f1000research.139309.2

**Published:** 2023-12-08

**Authors:** James Duminy, Alex Ezeh, Sandro Galea, Trudy Harpham, Mark R. Montgomery, J. M. Ian Salas, Daniela Weber, Amy Weimann, Danzhen You

**Affiliations:** 1 School of Geographical Sciences, University of Bristol, Bristol, England, UK; 2 African Centre for Cities, University of Cape Town, Rondebosch, Western Cape, South Africa; 3 Dornsife School of Public Health, Drexel University, Philadelphia, Pennsylvania, USA; 4 School of Public Health, Boston University, Boston, Massachusetts, USA; 5 London South Bank University, London, England, UK; 6 Department of Economics, Stony Brook University, Stony Brook, New York, USA; 7 Bloomberg School of Public Health, Johns Hopkins University, Baltimore, Maryland, USA; 8 International Institute for Applied Systems Analysis, Laxenburg, Lower Austria, Austria; 9 United Nations Children's Fund (UNICEF), New York, New York, USA

**Keywords:** emography, urban health, wellbeing, population, urbanization, cities

## Abstract

The focus is on the demographic drivers and demographic implications of urban health and wellbeing in towns and cities across the globe. The aim is to identify key linkages between demographic change and urban health – subjects of two largely disparate fields of research and practice – with a view to informing arguments and advocacy for urban health while identifying research gaps and priorities. The core arguments are threefold. First, urban health advocates should express a globalized perspective on demographic processes, encompassing age-structural shifts in addition to population growth and decrease, and acknowledging their uneven spatial distributions within and between urban settings in different contexts. Second, advocates should recognize the dynamic and transformational effects that demographic forces will exert on economic and political systems in all urban settings. While demographic forces underpin the production of (intra)urban inequities in health, they also present opportunities to address those inequities. Third, a demographic perspective may help to extend urban health thinking and intervention beyond a biomedical model of disease, highlighting the need for a multi-generational view of the changing societal bases for urban health, and enjoining significant advances in how interested parties collect, manage, analyse, and use demographic data. Accordingly, opportunities are identified to increase the availability of granular and accurate data to enable evidence-informed action on the demographic/health nexus.

## Key messages

• Demographic changes exert powerful forces on health and wellbeing within and between urban areas, and create new needs and challenges for health programming in all cities and regions.
• A nuanced, globalized demographic perspective can generate useful insights that will benefit theory and practice surrounding urban health and wellbeing.
• Improved urban health and wellbeing can help to enhance the positive effects (and alleviate the potentially negative effects) of demographic changes.
• The relationship between demographic and urban changes is dynamic and transformational.
• Political and policy debates around demographic change are remarkably diverse across country contexts.
• A narrow focus on population change, especially when framed in terms of ‘population control’, can be counterproductive when advocating for a renewed focus on demographic issues.
• The relationship between demographic changes, urban health, and economic development is a potential entry point for advocates of urban health and wellbeing.
• Demographic processes will shift the terms of the politics of urban health in ways that can be anticipated and planned for.

## Introduction: Demographic change and urban health

In this article we discuss the key mechanisms that connect demographic change to urban health and wellbeing, with a view to informing arguments and advocacy for urban health. We also identify key research gaps and priorities for an emerging demographic/health agenda centred on urban areas. We argue for a transformed approach to understanding and addressing the interface between demographic change and health in towns and cities. This approach should be based on a globalized recognition of the diverse, dynamic, and transformational demographic processes underway in different parts of the world. Within this perspective, we emphasize that one simply cannot think about demographic change and urban health with a view of urban populations as homogenous. Health in towns and cities is characterized by enormous heterogeneity within and between urban areas of different type and status ( Galea, Ettman and Vlahov, 2019). Consequently, we should understand how demographic processes are linked to the production of intra-city social and spatial inequities, alongside urban health outcomes at wider scales, and how demographic change can create opportunities for addressing such inequities and producing positive outcomes for urban health.

Understanding and addressing this interface requires bringing together or ‘linking’ two relatively discrete fields of inquiry and practice – demography and urban health – to forge a novel agenda for the delivery of sustainable and healthy cities. In doing so, we would underscore three points. First, an agenda to promote a productive relationship between urban health and demographic change may demand interventions that are not directly related to the health sector itself. Second, this agenda calls for detailed attention to the quality and availability of suitably disaggregated data, and the need for reform of existing data-gathering instruments. And third, the question of linking these two fields should also include a concern with the ‘means of implementation’ – the governance preconditions necessary to give effect to this agenda – if the potential of demographic change to improve the health and wellbeing of urban populations is to be realized.

The series of papers to which this article contributes addresses a range of themes. A general challenge here is to describe cross-thematic linkages (indicating systemic linkages and key leverage points within current debates) without diluting our central focus on demographic change. We have chosen to discuss the links between demographic change and *migration*, where migratory processes exert significant effects on how populations are changing (with direct consequences for urban health). We have also identified the links between demographic change and *climate change*, specifically where urban population change will exert a significant effect on emissions and on the vulnerability of city and town populations to extreme events and the effects of climatic change. These issues are covered in their own right in papers by Sa Machado *et al*. and Vardoulakis *et al*. in this collection (both forthcoming).

A principal theme in what follows has to do with the intersection of three spaces: the jurisdictions in which governmental units operate, the space where health and demographic forces are at play, and the spatial bins into which demographic and health data are collected. The lack of coherence in these spaces has given rise to the distinct terms ‘urban’ and ‘city’, which are often mistakenly viewed as synonymous. The differences between the two must be understood to appreciate the governance challenges that lie ahead.

Across national statistical offices, there is an unmistakable trend underway in the direction of what are often termed ‘statistical definitions of the urban’, by which urbanized areas are defined in terms of population density thresholds, contiguity criteria, and criteria on the total population size of candidate urban areas. The Degree of Urbanization estimates, developed by the European Commission and endorsed by the United Nations Statistical Commission, extend this general approach on a nearly global basis, thereby offering a new, globally-comparable synthetic approach to defining and measuring urbanized areas ( Dijkstra *et al.*, 2021). Statistical urbanization methods combine population census data for subnational administrative units with remote-sensing estimates of land covered by structures, with fine spatial detail needed in both of these dimensions. Hence this and other ‘statistical’ approaches depend crucially on the regular production of population censuses and the release of census detail at the level of small spatial units.

One of the many meanings of the word ‘city’, by contrast, is that of a unit of local governance, and specifically, municipal government. One of the merits of the Degree-of-Urbanization approach is that it highlights geographic spaces in which population is clustered in urban-like conditions. These dense clusters may, and generally do, span the jurisdictions of multiple local governments. The overlay of spatial population estimates, on the one hand, against the legal boundaries within which such governments are allowed to act, on the other, illustrates a central urban governance challenge with direct relevance to urban health promotion ( Montgomery, Pinchoff and Chuang, 2022).

Where health programmes and policies are concerned, further complications arise from the presence of multiple layers and units of government (which are especially evident in larger cities and towns) and the diversity of the private health sector in cities and towns irrespective of size. Sustained discussion of the demographic/health nexus, focused on cities and towns, is thus long overdue.

Another layer of complexity is added to this discussion when we consider ‘the urban’ in a more political and cultural register that is not limited to the geographic space or administrative jurisdiction of a city. Recognizing the distinction and dialectic between the material city and a socio-political sense of urbanism provides a useful perspective for the present topic. Here we recognize that demographic forces generate emergent political and cultural structures and phenomena affecting how claims are made on the city (including those related to urban health), and how resources are apportioned. Such claims shape the production of urban space, in both material and sociological terms. Those modes of production, in turn, condition the nature and distribution of demographic processes. Our discussion and arguments follow this view of urban areas, and urban health, as complex, emergent systems.

## Findings

### Demographic changes exert strong effects on urban health in all cities and regions

Demographic change does not refer only to changes in total population size. Rather, it encompasses a range of complex transformations including total population growth and decline, shifts in spatial distribution (across geographies and between urban and rural settings within countries), age-structural shifts (including ageing and youth ‘bulging’) and the evolution of family sizes and structures. These transformations have complex implications for formal healthcare provision and caregiving more generally, as well as for economic development, urban spatial transformation, and other factors, all of which will exert strong forces on urban health and wellbeing in future years.

Some demographic trends can be understood as global in scope. For example, nearly every country across the world is currently experiencing growth in both the size and proportion of its population of older people. Other trends are more specific to context. For instance, rates of population growth differ markedly across countries, regions, cities, income groups, and even by city neighbourhoods. Given this, how do (and will) demographic factors affect urban health and wellbeing? Here we briefly discuss these effects in relation to three key domains: population change, age-compositional change, and migration.

#### Population change

Future increases in the world’s population will take place almost exclusively in the cities and towns of low- and middle-income countries (LMICs) ( United Nations, 2019). This growth will be concentrated in sub-Saharan Africa and South Asia. In these regions, the urban proportion of the population will rise as urban population growth rates overtake rates of population growth overall. The resulting processes of urbanization, especially in poorer settings, often outpace the capacity of governments to make infrastructural investments that promote health and wellbeing in urban settings – including housing, water and sanitation, healthcare and education services. Consequently, increasing numbers of the urban population will be found in informal settlements and slums where health and wellbeing indicators are compromised ( Ezeh *et al.*, 2017; United Nations, 2019).

In some low- and middle-income countries (LMICs), high urban fertility rates linked to an unmet need for family planning ^1^, and which in some African settings will be sustained by stalls in urban fertility declines ( Sánchez-Páez and Schoumaker, 2022), are associated with health risks for individual women and children. These are expressed through, for example, resorts to unsafe abortion, perinatal health complications, and knock-on effects for reduced female employment as well as early childhood health and nutrition. In many LMICs, unmet need for family planning and resulting health risks often show significant intra-urban inequalities ( Duminy *et al.*, 2021). Meeting unmet need for family planning in urban areas would improve maternal and child health outcomes, facilitate urban fertility declines, and reduce gaps in the provision of urban services and housing in the longer term ( Ezeh, Kodzi and Emina, 2010).

Aside from the direct health and service implications of high urban fertility rates, population changes will affect health and wellbeing by aggravating or creating new vulnerabilities to climatic change. It is now increasingly critical for researchers and practitioners to understand the spatial distribution of urban population growth and its relationship to the impacts of climate change. Coastal zones, vulnerable to climate change effects including sea-level rise and flooding, already host very large urban populations. These areas tend to be more densely populated than inland areas and see higher rates of population growth ( MacManus *et al*., 2021). The next decades will see significant growth of the global population living in coastal areas at risk from sea-level rise and flooding, with the largest absolute growth in exposure to take place in Asia and the largest relative growth in Africa ( Merkens *et al.*, 2018). The urban health implications of these trends include sea-level rise that increases the salinity of groundwater, thereby threatening sources of drinking water and irrigation; the generation of epidemiological risks through the destruction of critical urban infrastructures and the spread of pathogens through flooding; and health-related issues arising from increased internal or international migration into urban areas driven by coastal disasters.

Cities are believed to be expanding spatially at rates faster than those at which their populations are growing. In some cases, this is linked to trends towards smaller household sizes. Aside from the well-documented links between urban sprawl, emissions and biodiversity loss, urban sprawl has implications for equity: low-density lifestyles and environments tend to exhibit stronger social inequalities ( Wei and Ewing, 2018). Sprawl also generates risks for public health and wellbeing through increased air pollution and traffic incidents, reduced physical activity, and threats to sources of drinking water and the availability of green spaces. Moreover, when coupled with inadequate land-use planning and agriculture and livestock intensification, urban sprawl increases the risk of zoonotic diseases emerging due to increased human exposure to biodiversity at the peri-urban interface. Urban population and spatial growth also exert strong effects (mostly indirectly, through consumption) on biodiversity loss and ecological destruction. Urban population growth driven by high fertility rates will therefore play a significant role in eroding the ecological basis for good human health and improved wellbeing in many urban settings.

Finally, there is now a resurgent debate on the role of demographic change within the generation of climatic risks (primarily through increased emissions) and within potential responses to the global climate emergency. This debate has taken on a far more nuanced form than previous neo-Malthusian arguments, emphasizing the dynamic relationship between changes in population size, structure, and consumption in place of a narrow focus on population growth and resource decline ( O’Neill *et al.*, 2010). This work highlights that implementing appropriate policies to satisfy unmet global demand for family planning and reproductive health services could create significant environmental co-benefits by lowering fertility rates. However, we currently lack a detailed understanding of precisely how or to what extent such policies, by reducing emissions, could exert positive effects on urban health and wellbeing. More generally, within this debate it should be recalled that the scale of overconsumption among high-income populations (and the danger that high consumption rates per capita will be replicated as other contexts develop economically) is substantially more significant, in terms of overall impacts, than resource use by low-income urban populations.

The growth of many urban populations, and the emergence of new health risks in expanding towns and cities, will place additional net demands on formal systems of healthcare provision in those settings. In contexts where this growth is driven by natural population increase, there will be increasing needs for healthcare services and infrastructures catering for growing populations of children and young adults. Where growth is driven by urban in-migration, the health needs of migrants, refugees and internally displaced persons will need to be accommodated and met (see Migration section below).

While population growth has historically preoccupied policy discussions, another important dimension of global demographic change is the trend towards population decline or decrease seen in many regions of the world, especially in the countries and cities of Europe, East Asia, and Latin America. Given that many of these countries are already highly urbanized, the implications of population decline and the age-structural changes it brings could have acute consequences for health and wellbeing, especially in the care economy and with respect to old-age support. These challenges will raise new urban infrastructural needs and will have to be met in the context of declining per capita tax bases. The full range of implications of population decline and ageing for urban health and wellbeing are not yet fully understood.

#### Age-structure change

Disease burdens vary across age groups, and often between urban and rural areas ( Montgomery *et al*., 2003). Understanding age structural changes of urban populations within and across countries will be key to effective planning and programming to improve health and wellbeing of urban populations. For example, children are most susceptible to vaccine-preventable infectious diseases, young people experience injuries and violence as leading causes of death, while non-communicable diseases tend to drive health and mortality among older populations. Policy and care responses to these disease burdens also vary. Immunization and nutrition programmes can improve health at young ages, policies on violence and injury prevention programmes can reduce the burden of injury-related morbidity and mortality, while non-communicable diseases (NCDs) require improvements in healthcare service delivery, long-term care, and health promotion and prevention programmes. Non-health sector interventions (including infrastructural interventions targeting green/public space, transport, water and sanitation, housing, and so on) can also help address these burdens but may require different thinking and approaches for various groups and contexts. Ultimately, understanding the current age structure of a city, town, or urbanized area and how it is changing is key to formulating responsive approaches for health and wellbeing. When it comes to urban health, one size does not fit all.

As life expectancy and urbanization increase, many countries and regions can expect to see a growing proportion of their older populations (aged 65 years or more) living in urban rather than rural areas. Where these populations live, and the specific kinds of physical and social infrastructures accessible to them, will decisively shape their health outcomes ( Cagney, 2019). To pick just one increasingly important example, a specific urban health concern linked to these trends is the higher vulnerability of older people to the risk of heat stress in urban environments.

In some settings, the health needs of older people may be relatively well understood and represented. However, in some Asian and African contexts, where many people maintain dual residences in both urban and rural areas, we may not have a sufficient understanding of how older populations experience and manage health risks in urban settings (and for what outcomes), mediated as they are by the dynamics of kinship, culture, village networks and intergenerational mobility and care ( McQuaid *et al.*, 2021). Meanwhile, in higher-income settings, we arguably require a better understanding of how ‘healthy ageing’ in urban areas will be mediated by the wider economic and social forces shaping urban transformation. These include fiscal austerity, changing housing markets, and the privatization of urban space ( Buffel and Phillipson, 2016).

Having increasing shares of older populations living in urban areas will boost demand for the provision of long-term care from formal healthcare systems, placing additional stress on available public finances and capacity. While intergenerational care provision is currently a major resource for the long-term care of older people in many settings, lower fertility rates and changing family structures in urban areas may lead people to resort increasingly to formal systems of long-term care. Yet in under-resourced contexts, where formal healthcare systems will struggle to meet growing demand, many vulnerable urban residents – like chronically-ill single older people – will continue or be forced to rely on informal care networks, support for which should remain an imperative. In sum, how demographic change will affect the demand for and provision of care – both formal and intergenerational – in diverse urban settings remains a key knowledge agenda item, particularly in under-researched LMIC contexts.

#### Migration

While the majority of urban growth in LMICs is contributed by natural increase, urban in-migration remains a key trend and driver of that growth ( Duminy *et al.*, 2021). However, the connections between migration, demographic change and urban health are currently not adequately understood. Migration may contribute to the production of intra-urban inequities as new arrivals are excluded from, or cannot afford, adequate educational and healthcare services in their city and town destinations ( Galea, Ettman and Zaman, 2022). Whether migration can create opportunities to address those inequalities is less clear.

The peak ages of internal migration range from the late teens (especially for girls) to the mid-twenties in most populations, thus spanning ages at which formal education is being completed to ages well into the years of marriage and reproduction. As a result, urban moves often situate these young people in environments in which potentially supportive educational and health resources are more plentiful than they tend to be in rural areas; yet there is certainly no guarantee that recent urban in-migrants will have access to such resources ( Montgomery *et al.*, 2016). Moreover, and contrary to common belief, urban-to-urban migration now (apparently, and no doubt with much variation across countries) rivals rural-to-urban migration in scope. Little is known about the implications of this shift, but the implication is that recent migrants may not be as uninformed about city life and urban resources as many have commonly thought.

Migration can also influence the processes underlying demographic changes. Most research confirms that rural-urban migration has a downward effect on fertility rates overall, alongside positive effects on contraceptive use. It seems that migrants adapt to their new urban conditions and assume behaviours that are prevalent among permanent or more established populations ( Montgomery *et al.*, 2003). Despite a considerable literature on migration and health, we still lack an adequate understanding of how various migration patterns, including the movement of people within and between urban areas – some of which will be driven, accelerated, and reshaped by climate change and natural disasters – impact both demographic and urban change in LMICs, including factors related to urban health and wellbeing ( Galea, Ettman and Zaman, 2022).

### Urban health advocates should develop and follow a transformed approach to understanding the relationship between demographic change and urban health

#### A globalized perspective on demographic change

Discussions of the demographic/health nexus within urban studies (that is, research from urban subdisciplines of geography, planning, sociology, political science, and so on) tend to focus on processes of rapid urban population growth or urbanization driving the historically unprecedented emergence of ‘megacities’, urban sprawl, the formation of slum-like or informal settlements, and growing risks of infectious disease ( Duminy, 2023). By contrast, in the field of ‘urban health’ population ageing is occasionally presented as the most important demographic shift of this century, even if this trend has yet to manifest itself across sub-Saharan Africa and South Asia ( Galea, Ettman and Vlahov, 2019). Both emphases are valid, and should form part of a broader perspective on the demographic/health nexus.

We aspire to a globalized perspective on demographic changes. Demographic changes should be seen as including complex processes related to population change (growth and decrease), age-structural shifts, and family and household structures. These transformations are and will be unevenly distributed geographically, between regions, between types/categories of urban areas, and between different areas within towns and cities. Health programming and research should take greater account of this diversity of demographic characteristics and trends ( You *et al.*, 2021).

#### A dynamic and transformational perspective on demographic and urban change

In line with a view of urban areas as dynamic and emergent entities, we emphasize that demographic changes will help to drive fundamental changes to the structure of towns and cities, as well as how they grow and function. Cities and towns will not simply be larger or smaller (whether in terms of population size or spatial extent) as a result of demographic changes. Rather, these changes will exert strong transformational effects on how and where people live, on how they organize politically, on how they see themselves subjectively and behave culturally, and on how cities function economically. A holistic approach to urban health and wellbeing could assist in harnessing these transformative demographic forces to promote positive social, economic, and political outcomes.

## Discussion: Policy and political debates and implications

Demographic change will have implications for the political basis for addressing urban health and wellbeing in future decades. However, political and policy debates surrounding ‘demographic change’ signal different things in different contexts. In the United States, the question of demographic change may be equated with shifts in the racial and cultural composition of the national population (in part linked to international in-migration). In some LMICs, it may be equated with rapid urban population growth and urbanization (often assumed to be driven primarily by rural-urban migration), associated with the extension of urban poverty and production of slum-like and/or informal settlements. In some upper middle-income countries (UMICs) and high-income countries (HICs), the core issues may be population ageing and (urban) population decrease. In certain settings, addressing a demographic agenda will attract controversy, in others not. Given this variability, here we consider the following questions:

• What are the political challenges or risks of promoting a focus on the relationship between demographic change and urban health?
• What are key policy debates that bear upon the relationship between demographic change and urban health?
• How is demographic change altering global, national, and urban policy landscapes?
• What implications will these factors have for urban health policy and practice over the coming decade?

In answering the first two questions, we should learn from the political processes surrounding recent examples of urban health policymaking and programming. Examples include the Ethiopia Urban Health Extension Programme, launched in 2009, India’s National Urban Health Mission, launched in 2013, and the 2020 Bangladesh National Urban Health Strategy. There is a general need for historically informed research attuned to the political preconditions and factors affecting the uptake (or not) of demographic problems within an urban health frame, in a variety of contexts.

### The spectre of population control

Until the last decade of the twentieth century, policy discussions of demographic change often were dominated by the question of whether population growth is a problem for economic development and/or environmental sustainability. The debate remains controversial, especially among non-demographic experts, and advocating a demographic agenda that draws attention to negative effects of high fertility rates in LMICs or pro-natalist policies in higher-income contexts risks attracting accusations of neo-Malthusianism, racism, and/or sexism. In population policy and research circles, these concerns took expression in the 1994 Cairo Programme of Action. This was a landmark event that decisively shifted emphasis from macro-level rationales to justifications based on individual rights, including the right to be free from coercion, bringing welcome attention to women’s reproductive health. Today, many political economists and environmentalists would add that a focus on ‘population control’ distracts from the systems of production, consumption, and inequality that underpin climatic and environmental problems. Nevertheless, there remains considerable resistance to demography-facing interventions such as family planning programmes as routes to sustainable and productive futures.

Demographic changes unfold over a complicated political terrain, but some of the reluctance to engage seen outside the population field could be addressed by:

• Emphasizing the potential impacts on urban health of high unwanted fertility rates (and unmet need for family planning) in some settings alongside the health implications of urban population ageing and decline in others. Here it should be recognized that high fertility rates may co-exist with a growth in the absolute number of older people within the same population.
• Noting that urban levels of unmet need for contraception are often significant, even in seemingly well-resourced cities and towns. The implications of unmet need and unwanted fertility, rather than high fertility as such, should be the entry point and principal theme for a discussion of demographic change and urban health. While we cannot ignore the discomfort that some policymakers experience in addressing contraception, pointing to (scattered) estimates of the incidence of induced abortion in LMIC cities and towns may help.
• Highlighting the systemic links between demographic changes (with respect to fertility rates and age structures), diverse migration patterns, and implications for conflict/security and economic change at a range of geographic and temporal scales.
• Refocusing the energy of current priorities towards ensuring that past mistakes do not reoccur. For instance, it is conceivable that the current push to incentivize childbearing in low-fertility countries may be seen as coercive in future decades as they generally involve financial incentives that work most effectively among the poor.

### Demographic change, economic development, and health

There is little debate that population growth, decline, and age-structure changes exert strong effects on economic development from the local to the global scales, with potentially significant implications for health and wellbeing. But less consensus exists on the direction and strength of any one specific effect. Much depends on the trade-offs and potential complementarities among fertility, investments in human capital per child and per student, and investments in physical capital, all of which influence the course of economic growth.

Countries with growing populations of children and/or older persons living in towns and cities are likely to see additional demands placed on state and family budgets. This will likely strain the public finances and services that can be directed to education, systems of healthcare and social security, critical urban infrastructures, and other wealth-generating interventions. High levels of state spending on young-age and older-age dependants have the potential to ‘squeeze’ the living standards of working-age populations ( Mason and Lee, 2022).

However, demographic changes can also generate policy opportunities to improve healthcare and the health of urban populations. To some, ‘population age structure and health status [are] key demographic determinants of economic progress’ ( Bloom and Canning, 2007). The concept of ‘demographic dividends’ and ‘windows of opportunity’ has proven influential in policy circles, often inspired by the astonishing growth (linked to rapid demographic transitions) achieved in East Asian economies such as South Korea and Thailand since the 1970s. Currently, these discussions concentrate on the potential for economic growth in contexts of Latin America, South and South-East Asia, and sub-Saharan Africa.

The windows of opportunity can take several forms (following Fried, 2016; World Bank, 2016):

• First, mortality decline in its initial stages improves young-age survivorship, leading to larger-than-expected cohorts of surviving children. In time this results in a larger-than-expected cohort of labour market entrants, which may find expression either in a boost in productivity due to the bump in the aggregate labour force, or in a logjam in sorting the new labour market entrants into productive employment.
• Second, as fertility rates drop in response to the initial mortality decline, and young-age dependency ratios decrease, this potentially frees resources to improve human capital investments per child, whether made by the family or the state or both. Economists are increasingly interested in the long-term productive payoffs that result from parental-time-intensive, interactive modes of early childcare and education, which are modes of parental and school-system care that are facilitated by low-fertility environments. Such life-course perspectives apply to the promotion of urban health. ^2^
• Third, as age structure shifts lead to an expansion of production and resources, a second window of opportunity can open as financial instruments and markets mature and stocks of savings grow, making possible increased investments in human and physical capital.
• Fourth, when the benefits for society borne of the social and economic capital of older people are realized, for which health-promoting investments over the life-course of individuals are a critical precondition, another window of opportunity may emerge. This window, associated with improved old-age survivorship, is highly conditional but would likely involve systemic changes that facilitate financial savings specifically to support a lengthening and active period of older age.

Meanwhile, the economic implications of ageing populations and population decrease have also attracted public and policy attention in diverse contexts including Singapore, Lebanon, South Korea, Australia, Vietnam, Italy, Russia, and some countries in Eastern European (for example, Bulgaria) and Latin America (for example, Chile and Costa Rica). Population ageing is often portrayed as being detrimental for economies owing to the reduced size of productive workforces, limited tax revenues, and increased (health)care and pension costs. Yet those relationships are not immutable recent research shows that declines in economic growth associated with population ageing can be moderated if older populations are in relatively good health ( Cylus and al Tayara, 2021).

Whether low fertility rates and population ageing are a problem for countries depends on a complex interplay of factors including age-related patterns of income from labour, consumption levels, and the nature and extent of intergenerational transfers of wealth and care ( Lee, Mason and members of the NTA Network, 2014). We can understand these relationships with the aid of two variables. A high *fiscal support ratio* (where a society enjoys a greater ratio of taxpayers to beneficiaries) tends to be favourable for public finances, allowing provision of more benefits at each age, lower taxes, a smaller budget deficit, or a combination of those three factors. A society with a higher *support ratio* (having more earners relative to consumers) means that each of its members enjoys proportionally greater availability of resources. That availability allows for more consumption, boosted savings and investments, or some combination of the two ( *ibid.*). If either or both ratios are relatively low in a society, the sustainability of health and welfare systems are potentially threatened.

The extension of years of good-quality life is in itself a profoundly beneficial advance. There is a risk of being too narrow in framing economic development in conventionally economic, ‘monetized’ terms. That being said, the conditions under which old-age groups are adequately supported and enjoying good-quality lives is due to prior investments made in the human capital of all population groups, increases in the physical capital with which the labour force works, and rates of innovation and technological progress.

A key ongoing debate centres on the fundamental drivers of declining fertility rates, and hence on the best mechanisms to reduce fertility rates and deliver associated benefits. Recently researchers have argued that investments in human capital such as education are more significant in delivering the demographic dividend than changing age structures ( Lutz *et al.*, 2019). There is also some debate concerning the causes of stalls in the fertility declines of African countries and urban areas ( Schoumaker, 2019). At minimum, these debates imply that an advocacy agenda centred on the urban health/demographic nexus should be careful to encompass and demonstrate the links between demographic shifts, wider political and economic transformations, and necessary investments in human capital to ensure that the positive effects of these shifts are enhanced, or their negative effects are alleviated.

Much of the work assessing the economic dynamics and impacts of population change focuses on the national or regional level and has yet to be downscaled to urban and intra-urban areas. How such factors could or will affect urban health and wellbeing in different contexts has not yet emerged as a specific topic of research interest. We could reasonably hypothesize that investments in urban health and wellbeing could play an important role in promoting the positive effects and alleviating the negative effects of demographic changes in towns and cities. No studies have assessed the specific extent to which urban health investments might do so, or how such investments should be optimally targeted in space and time.

### Demographic change and shifting policy/practice landscapes

Changing age profiles in societies will shift the political influence of various demographic groups; some groups will be better positioned than others to make claims on public and private institutions for the provision of social security, healthcare, and other health-promoting services or infrastructures. Alternatively, this political influence may be used to resist changes that would promote the health of other urban groups or urban populations more generally.

Demographic processes will ultimately shift the terms of electoral and patronage politics, and by implication the politics of urban health, in all settings. For example, the political implications of large (often unemployed and deprived) youthful urban populations in African settings is well documented. In India, the politics of the youth attracts public attention, while the significance of youth-led political mobilization has been noted further afield in relation to the Occupy movements of North America, the ‘Arab Spring’ uprisings, the 15-M Spanish anti-austerity movement, and the 2019–20 Hong Kong protests, among other events. As political attention shifts towards the demands and aspirations of urban groups in many contexts, particularly less-urbanized LMICs, we can expect renewed accusations of ‘urban bias’ to emerge and strengthen alongside reactionary political coalitions.

The growing political influence of older populations in North American and European contexts is also much discussed ( Greer *et al.*, 2021). Countries like Singapore, South Korea, Japan, and Russia have adopted pro-natalist policies to compensate for low fertility rates and population ageing. The implications of pro-natalist policies for future consumption, emissions, and climatic/environmental change have not attracted much critical debate. Many countries resorting to pro-immigration policies have experienced ‘nativist’ political backlashes.

Moreover, demographic changes will colour the nature, extent and characteristics of social movements related to urban health. How this happens should be considered both in terms of the *participants* of social movements – how they shape the goals, ideologies, and tactics of their movements – and in terms of how the demographic characteristics of *society* more broadly influence such factors ( Goldstone, 2015). A paper by Thomas *et al*. in this collection (forthcoming) examines social justice and equity movements in the context of urban health.

### What insights for urban health does a demographic perspective offer?

The emergence of an influential and widespread ‘age-friendly cities and communities’ movement over the past several decades has served as a helpful extension of earlier ‘healthy city’ concerns and programmes ( De Leeuw, 2017). In particular, the age-friendly movement has:

• Drawn attention to the specific health and wellbeing needs of certain cohorts, the diversity of settlement and community settings in which age-structural changes take place (including small towns, suburbs, and secondary cities) and the extent to which these settings promote and enhance health throughout one’s life;
• Mobilized concepts and frameworks such as the ‘life-course approach’ and ‘person-environment fit’ ( WHO, 2015). The notion of the ‘life course’ draws attention to the wide range of protective and risk factors that interplay in health and wellbeing over the lifespan. The notion of ‘person-environment fit’ emphasizes the health risks/outcomes that arise from the dynamic interactions of particular demographic groups and the specific environments they inhabit during the life course; and
• Targeted particular social groups as the ‘entry point’ for thinking about analysis and intervention (rather than using the health sector as the entry point), recognizing the social determinants of health, and potentially extending urban health programmes to a wider range of problem definitions and solutions beyond a biomedical or health sciences model of health promotion.

More generally, a demographic perspective is useful in highlighting issues of temporality and the necessity of having available and accurate data. That is, a demographic perspective reveals the medium- and long-term structural transformations that will influence how cities and urban health change (including age-structural shifts and related disease burdens), how much money is available to states and societies to invest, how local and national governments plan, and when and where money should be invested. Consequently, promoting urban health and wellbeing should involve anticipating both short-term and multi-decadal demographic changes and their geographic distributions, while planning and investing in health-promoting initiatives through spatial and temporal targeting, including through investments and practices that are not necessarily directly related to the health sector (e.g. urban planning or urban infrastructure finance and management). A demographic perspective therefore highlights the importance of having data that are accurate, recent, and representative of the population, and of collecting longitudinal data that enable the separation of correlation from causality, which is important when attempting to influence policymakers and decision-makers.

The Global Monitoring Report 2015/2016 ( World Bank, 2016) produced a new global typology of countries that ties demographic change to development potential. The typology recognized the various pathways through which demographic change affects the prosperity of nations, enabling the disaggregation of policy priorities and recommendations according to the position occupied by countries within this typology. Countries were grouped into pre-demographic-dividend, early dividend, late dividend, and post-dividend categories.

A holistic approach to urban health and wellbeing could make use of a similar typology that is specifically tailored towards urban areas. Urban health programmes in different contexts could be framed as helping cities to progress positively through the stages of the demographic-developmental transition. Such programmes would take a long-term view and recognize that initial policy priorities (such as increasing access to family planning in pre-dividend cities) may need to give way to other priorities and interventions as cities ‘mature’ (for example, health policies that promote and protect female labour force participation in an early-dividend city). In the same way that a life-course approach draws attention to the ways in which different health risks and needs arise over the course of a lifespan, so a ‘city life-course approach’ would highlight that:

• Urban areas and populations will experience emergent health risks and disease burdens over time that are associated with demographic changes, with implications for where, how and on what money should be spent;
• Certain kinds of health interventions in the short-term and in the lives of children may be necessary to guarantee better health and wellbeing for urban populations in the longer-term as they develop and age; and
• Promoting better health and wellbeing of urban populations is a necessary condition for realizing the potential of cities as drivers of economic development (the ‘urban dividend’).

### Priority data and research

Our discussion has indicated a range of research areas and topics that demand further attention if we are to better understand and harness the relationship between demographic change and urban health. These should be seen as a complement to the set of global research priorities for urban health recently identified by the World Health Organization, which do not encompass demographic issues or processes ( WHO, 2022). Our priorities for data and research include investigation of:

• How different demographic trends may unfold simultaneously with varying implications for urban health – for instance, high rates of (urban) population growth in sub-Saharan Africa and South Asia alongside population decreases in Europe and East Asia;
• The health implications, linked to climate change, of having urban growth increasingly concentrated in coastal areas;
• The ways in which various densities of urban growth (realized as patterns of sprawl or compaction), in the context of climate change, will affect health through changing patterns of land use, biodiversity loss, the emergence of zoonotic diseases, and so on;
• How the age profiles of urban residents intersect with other factors and potential sources of vulnerability, such as socioeconomic status, social interaction/loneliness, and mobility and migration, throughout the life course.
• How changing age structures occasioned by fertility and mortality patterns will create differentiated disease burdens in urban areas – indicating the need to develop a typology of urbanization, population, and health dynamics and how these relate to urban health and wellbeing;
• The health of older people living in urban areas, not only in high-income urbanized contexts with ageing populations, but also in lower-income settings where social systems are shaped by patterns of migration and dual residence; and
• How demographic change will affect the demand for and provision of care in diverse urban settings, particularly in LMICs.

Addressing these research priorities will require considerable advances in the availability and quality of suitably disaggregated data. It is now clear that simple national disaggregation by urban and rural categories will not be sufficient for an accurate understanding of the interface between demographic change and urban health ( Duminy *et al.*, 2021). At minimum, researchers and policymakers require data revealing how demographic factors are distributed among different settlements and types/categories of urban areas, including intra-urban differences. Disaggregation by ‘slum’ and ‘non-slum’ categories (as in the Bangladesh Urban Health Survey programme), or by wealth index (as with India’s National Family Health Survey), provides a useful starting perspective on inequality that ideally would be refined through a focus on neighbourhood-level differences. Moreover, urban demographic data could be linked to climate projections to enable a better understanding of how different demographic cohorts living in towns and cities will experience emergent climatic risks such as increased heat stress.

There are new developments in the domain of spatial data and measurement that present opportunities to make the need to address the demographic/health interface in towns and cities to policymakers and decision-makers more compelling than it may have been in the past. For instance, the ‘Degree of Urbanization’ ^3^ measure, which is derived from satellite data and scheduled to be updated every two years, offers a way of approximating, with high specificity, where urban spatial growth is taking place and at least provides a proxy of where urban population growth is concentrated.

In terms of reforming related programmes and datasets, we have identified the following priority actions:

• The National Transfer Accounts (NTA) programme: This programme and conceptual framework has provided important insights into how population growth and changing age structures influence economic growth, gender and generational equity, public finance, and other important features of the macro-economy. However, it remains to be seen whether the NTA framework can accommodate the effects of demographic change on *urban* health investments and policies. We urge an extension of the NTA Network to incorporate urban/rural dynamics within each country it analyses.
• The United Nations Population Division: Data released in the *World Urbanization Prospects* series have not been disaggregated by age or sex, which makes it difficult to analyze urban demographic trends even at the national level. The UN’s companion series, *World Population Prospects*, addresses a range of demographic indicators, including sex and age, but the tabulations are not spatialized even into broad urban and rural categories. We recommend that the United Nations incorporate disaggregation by urban/rural, age, and sex in its reports and datasets.
• The two major household survey programmes – the Demographic and Health Surveys (DHS) and the Multiple Indicator Cluster Surveys (MICS) – have each placed over 300 surveys into the public domain, focusing principally on high-quality estimation of health and socioeconomic indicators for national and urban/rural strata as well as first-order administrative levels. In an urbanizing era, these programmes face a daunting new challenge: how to expand and reconfigure their sampling frames to enable detection of inequalities in health *within* cities and towns, ideally at the neighbourhood level. The DHS has taken the essential first step in this direction, by distributing the spatial coordinates of its survey clusters and thus enabling links to be made to other data sources for neighbourhoods, health service locations, schools, transport routes, nearby sites of environmental or health risk, and so on. Although these coordinates are currently released only in displaced form (shifted by as much as two kilometres of random error) to preserve respondent confidentiality, consideration is being given to shrinking the radius of displacement while minimizing disclosure risk, given the high population densities of cities and towns. We hope that the MICS will follow suit, and strongly recommend reforms to both programmes that enable within-city analyses and comparisons.
• National statistical offices: Support is required for national statistical offices to provide spatially-specific subnational disaggregated data from their population censuses, which is an essential and irreplaceable ingredient for the Degree of Urbanization method.

Beyond the reform of international programmes, there are opportunities to make use of supplementary or alternative sources of data to enhance understanding of intra-urban dynamics and differentials. One cost-efficient approach is for national demographic and health surveys to include a ‘booster’ sample of selected cities ( Gupta *et al.*, 2009).

A second approach would be for researchers of the demographic/health nexus to engage with an urban-focused citizen science movement. Technology-aided social or community-based mapping and census-taking offer a set of approaches with the potential to generate accurate local datasets that take account of local definitions and social dynamics, encourage community engagement, while promoting local ownership and coproduction of data and project interventions. There may be opportunities to work with and learn from urban-focused networks like Slum/Shack Dwellers International (SDI), which promote community-based enumeration activities in poor urban areas of LMICs, but not necessarily with an explicit or detailed health lens ( Patel *et al.*, 2012). Nonetheless, these initiatives provide granulated insights that could be linked to existing demographic and health datasets, or communities undertaking self-enumerations could be encouraged to collect data on appropriate demographic and health factors. For their part, such organizations would benefit from the skills and techniques of demographic and health experts with their methodological rigour and the quality of their datasets.

We have argued that an effective response to the demographic-health agenda in urban areas demands data that are available for most contexts, accurate, recent, and sufficiently spatially and demographically disaggregated. However, discussions of data reform often assume that policymakers and decision-makers will somehow have the appropriate professional and technical capacity to use these data to analyse and act on complex urban relationships. There may be a need for a specific research agenda that considers, for example, the kinds of intellectual, professional, and technical capacities that are required to adequately document, understand, and act on the relationships between demographic change and urban health and wellbeing. This kind of research agenda, which is concerned more with the ‘means of implementation’ than the substantive nature of the demographic-health interface, is all the more pressing given that the agenda addressed by this article calls for the ‘joining up’ of two relatively discrete fields of inquiry and practice: demography and urban health ( Harpham *et al*., 2021). In some cases, that process of ‘linking’ may require:

• The education and training of professionals who are legible across intellectual and technical domains, or at least able to converse with professionals drawn from fields different to their own;
• Leadership development programmes to build the capacity of local leaders to harness spatial-demographic data to solve problems related to urban health; or
• The creation of appropriate partnerships for data collection and management between levels or sectors of government that include but extend beyond health departments or health-focused research centres.

Consequently, there is a need to gather case studies of institutions or initiatives that have attempted to link the demographic and urban health (or perhaps other) fields through data practices, to build capacity to analyse complex urban problems, and to use the resulting information to shift the basis of policy- and decision-making. Examples include the work of the African Population and Health Research Centre, which has used longitudinal data on urban demographic and health change to influence policymaking in Nairobi and Kenya, or the Bloomberg Centre for Government Excellence at Johns Hopkins University, which supports and coaches local leaders to build data-driven approaches to urban governance.

## Conclusion

Diverse demographic changes will have significant and transformative impacts on urban health and wellbeing in all regions of the world, but the demographic/health nexus remains an under-appreciated interface of urban research and governance intervention. A view of this interface should recognize that, globally speaking, demographic changes take diverse forms and are unevenly spatially distributed within and between towns and cities. Demographic forces underpin the production of (intra) urban inequities in health, yet in their transformational nature also present opportunities to address those inequities. A demographic perspective highlights the need for a long-term multi-generational view of the changing societal basis for urban health, calling for significant advances in how interested parties collect, manage, analyse, and use data. Doing so will provide the intellectual and practical basis for an effective response to the urban health and wellbeing challenges of the twenty-first century.

## Data Availability

No data are associated with this article.

## Footnotes

^1^
The proportion of women who want to avoid or delay another birth but who are not using any method of contraception.

^2^
‘A life course approach posits that early experiences are deeply influential on our later behaviours and characteristics, including our health. This puts a particular premium on thinking about early life as a determinant of the health of populations’ ( Suglia, 2019). An increasingly influential body of academic research on the long-term economic benefits from early child development, led by James Heckman, is summarized in https://heckmanequation.org/.

^3^

https://ec.europa.eu/eurostat/web/degree-of-urbanisation/background
