# Supplementary material for: Demographic change and urban health: Towards a novel agenda for delivering sustainable and healthy cities for all
Source: F1000Res. 2023 Dec 8;12:1017. Originally published 2023 Aug 21. [Version 2] doi: 10.12688/f1000research.139309.2 (PMC10905150; doi:10.12688/f1000research.139309.2)
Supplement: Peer review history

# Reviewer report: Reviewer response for version 2

**DOI:** 10.5256/f1000research.159707.r228715

**Published:** 2024-02-27

**Authors:** Lorenzo Paglione

**Affiliations:** 1 University of Rome,, Rome,, Italy

Authors followed the indications relating to the first review.  An excellent systematization work that allows us to clarify numerous aspects and links between urban dynamics from the point of view of the relationship between urban-architectural and socio-economic and demographic determinants.

Is the review written in accessible language?

Yes

Are all factual statements correct and adequately supported by citations?

Yes

Are the conclusions drawn appropriate in the context of the current research literature?

Partly

Is the topic of the review discussed comprehensively in the context of the current literature?

Partly

Reviewer Expertise:

Environmental hygiene, social epidemiology

I confirm that I have read this submission and believe that I have an appropriate level of expertise to confirm that it is of an acceptable scientific standard.

# Reviewer report: Reviewer response for version 2

**DOI:** 10.5256/f1000research.159707.r228714

**Published:** 2024-01-22

**Authors:** Siddharth Agarwal, Kanupriya Kothiwal

**Affiliations:** 1 Urban Health Resource Centre, New Delhi, Delhi, India

**The authors have incorporated most of the suggestions given in the peer review dated 8 ^th^ November 2023. We urge the authors to consider including the undermentioned suggestions which have probably been missed for some reason.**

 Citing context relevant primary research will enhance the arguments in the manuscript: The authors are urged to consider providing findings from context appropriate examples from relevant primary research studies to strengthen the arguments stated in the manuscript. The section on “Population change”, where authors have given emphasis to climate induced migration, can benefit from providing examples such as those of cities in Bangladesh which are witnessing increased population not only owing to climate induced migration but also due to opportunities that cities present to migrants. (Adri & Simon, 2018 ^1^).

Is the review written in accessible language?

Yes

Are all factual statements correct and adequately supported by citations?

Partly

Are the conclusions drawn appropriate in the context of the current research literature?

Partly

Is the topic of the review discussed comprehensively in the context of the current literature?

Yes

Reviewer Expertise:

Urban demography, urban health, intra-urban disparities in health, housing characteristics, wellbeing

We confirm that we have read this submission and believe that we have an appropriate level of expertise to confirm that it is of an acceptable scientific standard.

## References

[1] : A tale of two groups: focusing on the differential vulnerability of “climate-induced” and “non-climate-induced” migrants in Dhaka City. *Climate and Development* .2018;10(4) : 10.1080/17565529.2017.1291402 321-336 10.1080/17565529.2017.1291402

# Reviewer report: Reviewer response for version 1

**DOI:** 10.5256/f1000research.152575.r206395

**Published:** 2023-11-08

**Authors:** Siddharth Agarwal, Kanupriya Kothiwal

**Affiliations:** 1 Urban Health Resource Centre, New Delhi, Delhi, India

We recommend adding a few factual statements which will enrich the manuscript:

**Examining differences in health investments within the Global South: **In the section “Discussion: Policy and political debates and implications” it will be worth highlighting the health investments made in different LMICs. In Southeast Asia and Africa, countries have invested in reasonably sound policies in the view of changing demographics and urbanisation though the implementation of these policies has been weak.

 These include:

1. **Country urban health policies/strategies**: (i) In 2013, India launched the National Urban Health Mission aiming to provide comprehensive last-mile health delivery to the poorest in urban areas. (MoHFW, Government of India, 2013). (ii) Bangladesh launched its National Urban Health Strategy in 2014 (MoHFW, Government of Bangladesh, 2020). Countries in Sub-Saharan Africa are steadily making progress towards policy investments in healthcare with a focus on nutrition and urban agriculture in urban areas. (Kimani-Murage & Wanjohi, 2020). (iii) Ethiopia’s Urban Health Extension Program which is being implemented in major urban areas will be vital to include in the policy section. (USAID, undated)
2. **Citing context relevant primary research will enhance the arguments in the manuscript: **The authors are urged to consider providing context appropriate examples from relevant primary research studies to strengthen the arguments. The section on “Population change” where authors have laid emphasis on climate induced migration can benefit from providing examples such as those of cities in Bangladesh which are witnessing increased population not only owing to climate induced migration but also due to opportunities that cities present to migrants. (Adri & Simon, 2018)
  Examples of climate induced migration from rural areas of India to cities will be valuable to mention in this section. Erratic rainfall, droughts are climatic factors that are adversely affecting agriculture and agriculture related wage-earning labour jobs in rural areas in many parts of India, forcing families to migrate to cities for livelihood. (Debnath & Nayak, 2022)
3. **Key examples of disaggregated urban data**: Similarly, in the section Priority data and research, the authors have highlighted the crucial importance of disaggregated demographic data particularly examining intra-urban differences. An example which can support this argument is the analysis of India’s National Family Health Survey (NFHS) by wealth-index that unmasks the disparities in Indian cities with respect to child and maternal health, provision for health care and housing conditions. (Agarwal, 2011) . Intra-urban differences in health conditions have been studied in Bangladesh in the form of Bangladesh Urban Health Survey 2013. (UHS, 2013).

**Recommendations for strengthening the conclusion:**

  

1. **Importance of community-level enumerations in informing data:** In identifying priority actions related to reforming datasets, the authors may consider laying emphasis on the challenges of gathering data in urban informal settlements in LMICs and significance of the initiatives that many community-based organizations and researchers are undertaking in pursuing community-driven enumerations to gather data. (Vlahov et al., 2011). These include enumerations by National Slum Dwellers Federation (NSDF) in Mumbai, India (Patel S et al., 2012) and Slum and Shack Dwellers International (SDI) in many African countries. (Kuffer et al., 2019; Lines & Makau, 2018).
2. **Suggestion with a key example of a low-cost method to utilize DHS for analyzing intra-urban differentials at city-level: **DHS can do a booster sample of select cities in a country to then enable use of city data for analysing intra-city disaggregation. An example of such a DHS exercise is available from NFHS 3 (India) where the health and wellbeing conditions of populations of eight cities was described in a dedicated report entitled “Health and Living Conditions in Eight Indian Cities” released in 2009. Being part of the country’s DHS, an approach of having an additional sample of select cities is a cost-efficient method of research in urban areas of a country. Cost efficient ways of obtaining urban data with disaggregation are even more crucial in contemporary times where international financial assistance is challenging to get than it was a couple of decades ago. (Gupta et al., 2009)

**Summary of the article**: The research topic is timely and crucial in the evolving spectrum of global urbanization. The authors have meticulously laid arguments using relevant literature on the importance of a) interplay of urban health with demographic processes; b) impacts of demographic changes on economic and political systems in cities; c) significance of a demographic perspective in understanding urban inequities and using data to enable evidence-informed action.

 Citations/References

 Adri, N., & Simon, D. (2018). A tale of two groups: focusing on the differential vulnerability of “climate-induced” and “non-climate-induced” migrants in Dhaka City. *Climate and Development*, *10*(4), 321-336. DOI 10.1080/17565529.2017.1291402

 Agarwal, S. (2011). The state of urban health in India; comparing the poorest quartile to the rest of the urban population in selected states and cities. *Environment and Urbanization*, *23*(1), 13-28. DOI: 10.1177/0956247811398589

 Bangladesh Urban Health Survey (2013). https://www.measureevaluation.org/publications/tr-15-117

 Debnath, M and Nayak, DK (2022) Assessing drought induced temporary migration as an adaptation strategy: evidence from rural India. Migration and Development 11(3): 521–542 DOI 10.1080/21632324.2020.1797458

 Gupta, K., Arnold, F., & Lhungdim, H. (2009). Health and living conditions in eight Indian cities. National Family Health Survey (NFHS-3), India, 2005–06. *Mumbai: International Institute for Population Sciences*. https://rchiips.org/nfhs/urban_health_report_for_website_18sep09.pdf

 Kimani-Murage, E., & Wanjohi, M. N. (2020). *Urban Health Assessment: Nutrition and Water, Sanitation and Hygiene (WASH) Challenges Facing Children and Adolescents in Urban Slums in Nairobi*. African Population and Health Research Center. http://www.jstor.org/stable/resrep26381

 Kuffer, M., Persello, C., Pfeffer, K., Sliuzas, R., & Rao, V. (2019, May). Do we underestimate the global slum population?. In *2019 Joint Urban Remote Sensing Event (JURSE)* (pp. 1-4). IEEE. DOI 10.1109/JURSE.2019.8809066

 Lines, K., & Makau, J. (2018). Taking the long view: 20 years of Muungano wa Wanavijiji, the Kenyan federation of slum dwellers. Environment and Urbanization, 30(2), 407-424. https://doi.org/10.1177/0956247818785327

 Ministry of Health and Family Welfare, Government of India. National Urban Health Mission (2013) https://nhm.gov.in/index1.php?lang=1&level=1&sublinkid=970&lid=137

 Ministry of Health and Family Welfare, Government of the People’s Republic of Bangladesh. National Urban Health Strategy 2020. https://www.nnsop.gov.bd/storage/files/file-2023-08-29-64ed92c16323d.pdf

 Patel, S., Baptist, C., & D’Cruz, C. (2012). Knowledge is power – informal communities assert their right to the city through SDI and community-led enumerations. Environment and Urbanization, 24(1), 13-26. https://doi.org/10.1177/0956247812438366

 Vlahov, D., Agarwal, S.R., Buckley, R.M. *et al.* Roundtable on Urban Living Environment Research (RULER). *J Urban Health* 88, 793–857 (2011). https://doi.org/10.1007/s11524-011-9613-2

 USAID (undated). Ethiopia’s Urban Health Extension Program. https://publications.jsi.com/JSIInternet/Inc/Common/_download_pub.cfm?id=22119&lid=3

Is the review written in accessible language?

Yes

Are all factual statements correct and adequately supported by citations?

Partly

Are the conclusions drawn appropriate in the context of the current research literature?

Partly

Is the topic of the review discussed comprehensively in the context of the current literature?

Yes

Reviewer Expertise:

Urban demography, urban health, intra-urban disparities in health, housing characteristics, wellbeing,

We confirm that we have read this submission and believe that we have an appropriate level of expertise to confirm that it is of an acceptable scientific standard, however we have significant reservations, as outlined above.

## References

[1] : A tale of two groups: focusing on the differential vulnerability of “climate-induced” and “non-climate-induced” migrants in Dhaka City. *Climate and Development* .2018;10(4) : 10.1080/17565529.2017.1291402 321-336 10.1080/17565529.2017.1291402

[2] : The state of urban health in India; comparing the poorest quartile to the rest of the urban population in selected states and cities. *Environment and Urbanization* .2011;23(1) : 10.1177/0956247811398589 13-28 10.1177/0956247811398589

[3] Bangladesh Urban Health Survey.2013; Reference source

[4] : Assessing drought-induced temporary migration as an adaptation strategy: evidence from rural India. *Migration and Development* .2022;11(3) : 10.1080/21632324.2020.1797458 521-542 10.1080/21632324.2020.1797458

[5] : Health and living conditions in eight Indian cities. National Family Health Survey (NFHS-3), India, 2005–06. *Mumbai: International Institute for Population Sciences* .2009; Reference source

[6] : Urban Health Assessment: Nutrition and Water, Sanitation and Hygiene (WASH) Challenges Facing Children and Adolescents in Urban Slums in Nairobi. *African Population and Health Research Center* .2020; Reference source

[7] : Do we underestimate the global slum population?. In 2019 Joint Urban Remote Sensing Event (JURSE) (pp. 1-4). *IEEE* .2019; 10.1109/JURSE.2019.8809066 10.1109/JURSE.2019.8809066

[8] : Taking the long view: 20 years of Muungano wa Wanavijiji, the Kenyan federation of slum dwellers. *Environment and Urbanization* .2018;30(2) : 10.1177/0956247818785327 407-424 10.1177/0956247818785327

[9] : National Urban Health Mission.2013; Reference source

[10] : National Urban Health Strategy.2020; Reference source

[11] : Knowledge is power – informal communities assert their right to the city through SDI and community-led enumerations. *Environment and Urbanization* .2012;24(1) : 10.1177/0956247812438366 13-26 10.1177/0956247812438366

[12] : Roundtable on Urban Living Environment Research (RULER). *J Urban Health* .2011;88(5) : 10.1007/s11524-011-9613-2 793-857 10.1007/s11524-011-9613-2 21910089 PMC3191208

[13] : Ethiopia’s Urban Health Extension Program. Reference source

# Reviewer report: Reviewer response for version 1

**DOI:** 10.5256/f1000research.152575.r212320

**Published:** 2023-10-26

**Authors:** Lorenzo Paglione

**Affiliations:** 1 University of Rome,, Rome,, Italy

I believe that the text is an excellent contribution to the general discussion on the central issue of demography in urban contexts. However, some clarifications are probably necessary.

 Introduction: regarding the concepts of "urban" and "city", the distinction is perhaps made in a simplistic way, using fundamentally statistical tools. In this sense, the literature in the urban planning field and the so-called "critical urban studies" refers in particular to a more complex vision, which questions forms of dualism between material (through the Latin word urbs) and immaterial (through the Latin word civitas) . This adds, together with the very concept of irreducible dualism between the physical city and the city of social (and political) relations, a further level of complexity, which in the case of the discussion of this article on the deomographic theme, I believe is necessary.

 Findings - population change: regarding this paragraph, I think it is useful to specify, at the end, how there is not only the need for formal health systems, but also for informal support networks, in particular for that segment of the population, which is expanding, with socio-health fragility ( for example chronically ill single elderly people, or single-parent families with disabilities). In this sense, under penalty of collapse - or in any case the need for unlimited resources - the health service cannot also make up for the shortcomings of a widespread "care society", which also involves a strengthening of informal care processes.

 Demographic change, economic development, and health: regarding this paragraph I believe it is useful to further underline the link between the working active population, general taxation and sustainability of health services and welfare systems, and not only through the productivist vision of World Bank. 

 This relationship is central and cannot be addressed with just a few general phrases.

 Priority data and research: I believe that in this sense the link between climate change, loss of biodiversity and human health must be made explicit from a one health perspective (zoonoses are also dependent on climate change, which does not only concern the rising sea levels, but also extreme climatic phenomena, such as heat islands, which require careful evaluation in terms of available data and the ability to read contexts at a very high level of detail).

 In general: I believe we need to improve the connections between the parties. A common thread could be that of the intersectional approach and linked to the concept of vulnerability: the elderly person is vulnerable if alone or of a low socioeconomic class, as is the migrant, but above all the level of vulnerability (and also the vulnerability factor) changes during the life trajectory. Perhaps the possibility of examining these aspects in greater depth should be considered.

Is the review written in accessible language?

Yes

Are all factual statements correct and adequately supported by citations?

Yes

Are the conclusions drawn appropriate in the context of the current research literature?

Partly

Is the topic of the review discussed comprehensively in the context of the current literature?

Partly

Reviewer Expertise:

Environmental hygiene, social epidemiology

I confirm that I have read this submission and believe that I have an appropriate level of expertise to confirm that it is of an acceptable scientific standard, however I have significant reservations, as outlined above.
